# Concurrent Chemotherapy for T4 Classification Nasopharyngeal Carcinoma in the Era of Intensity-Modulated Radiotherapy

**DOI:** 10.1371/journal.pone.0119101

**Published:** 2015-03-06

**Authors:** Cai-neng Cao, Jing-wei Luo, Li Gao, Jun-lin Yi, Xiao-dong Huang, Kai Wang, Shi-ping Zhang, Yuan Qu, Su-yan Li, Jian-ping Xiao, Zhong Zhang, Guo-zhen Xu

**Affiliations:** 1 Department of Radiation Oncology, Cancer Hospital, Chinese Academy of Medical Sciences, Peking Union Medical College, Beijing, China; 2 Department of Radiation Oncology, Zhejiang Cancer Hospital, Hangzhou, China; IRCCS National Cancer Institute, ITALY

## Abstract

**Objective:**

To evaluate concurrent chemotherapy for T4 classification nasopharyngeal carcinoma (NPC) treated by intensity-modulated radiotherapy (IMRT).

**Methods:**

From July 2004 to June 2011, 180 non-metastatic T4 classification NPC patients were retrospectively analyzed. Of these patients, 117 patients were treated by concurrent chemoradiotherapy (CCRT) using IMRT and 63 cases were treated by IMRT alone.

**Results:**

The median follow-up time was 58.97 months (range, 2.79–114.92) months. For all the patients, the 1, 3 and 5-year local failure-free survival (LFFS) rates were 97.7%, 89.2% and 85.9%, regional failure free survival (RFFS) rates were 98.9%, 94.4% and 94.4%, distant failure-free survival (DFFS) rates were 89.7%, 79.9% and 76.2%, and overall survival (OS) rates were 92.7%, 78.9% and 65.3%, respectively. No statistically significant difference was observed in LFFS, RFFS, DFFS and OS between the CCRT group and the IMRT alone group. No statistically significant difference was observed in acute toxicity except leukopenia (p = 0.000) during IMRT between the CCRT group and the IMRT alone group.

**Conclusion:**

IMRT alone for T4 classification NPC achieved similar treatment outcomes in terms of disease local control and overall survival as compared to concurrent chemotherapy plus IMRT. However, this is a retrospective study with a limited number of patients, such results need further investigation in a prospective randomized clinical trial.

## Introduction

Concurrent chemoradiotherapy had become the standard treatment regimen of non-metastatic T4 classification nasopharyngeal carcinoma (NPC) for the most definite survival benefit [1–3]. However, most of these evidences were based on the non-intensity-modulated radiotherapy (IMRT) technique. Compared with 2D-conventional radiotherapy, IMRT could improve overall survival and local-recurrence free survival, especially in late-stage NPC patients [4].

Radiotherapy of T4 classification NPC is one of the greatest challenges for high tumor load and proximity of critical structures such as the spinal cord and brain stem. Our initial clinical experience [5] indicated that 2-year local failure-free survival rate and 2-year overall survival rate of T4 classification NPC treated by IMRT were 82.1% and 82.5%, respectively. Up to date, the role of concurrent chemoradiotherapy (CCRT) for NPC in the era of IMRT is unknown. The aim of this study is to evaluate concurrent chemotherapy for T4 classification NPC treated by IMRT.

## Methods and Materials

### Patients and patient workup

This study was approved by the independent ethics committee, Cancer Hospital, Chinese Academy of Medical Sciences to identify the patients diagnosed with NPC in our center. Because this study was a retrospective study, consent was not obtained and patient records were anonymized and de-identified prior to analysis.

Inclusion criteria were as follows: 1) histologically confirmed NPC by biopsy, 2) no evidence of distant metastasis, 3) Karnofsky performance score ≥70, 4) receiving radical IMRT or concurrent chemotherapy (Cisplatin, 30 mg/m2/w) at initial diagnosis, 5) no neoadjuvant or adjuvant chemotherapy, 6) no pregnancy or lactation, and 7) no previous malignancy or other concomitant malignant disease.

Between July 2004 and June 2011, 180 NPC patients who met all of the criteria were retrospectively analyzed. Of these 180 NPC patients, 117 patients were treated by CCRT using IMRT and 63 cases were treated by IMRT alone. The pretreatment workup included a complete history and physical examination, liver and renal biochemical analysis, complete blood cell count, chest X-ray, fiber-optic nasopharyngoscopy, magnetic resonance imaging (MRI) of the head and neck, bone scintigraphy, ultrasonography of the abdominal region, and dental check. In addition, thoracic computed tomography (CT) scan was required to be performed on N3 staging patients. All patients underwent disease staging using the AJCC 2010 staging system. The clinical characteristics are listed in Table 1.

**Table 1:** Patient characteristics.

Characteristic	CCRT group(N = 117)	IMRT alone group(N = 63)	*P*
**Gender**			
Male	85	44	.690
Female	32	19	
**Age (yr)**			
Median	47	54	.054
Range	12–70	14–77	
**Pathology classification**			
Keratinizing	1	0	1.000
Non-keratinizing	116	63	
**N category**			
N0	11	6	.885
N1	34	23	
N2	66	32	
N3a	2	1	
N3b	4	1	
**Metastasis to retropharyngeal lymph nodes**			
Yes	93	49	.789
No	24	14	
**Involvement of cranial nerves**			
Yes	35	13	.179
No	82	50	
**Boost**			
IMRT	18	9	.758
SBRT	11	7	

Abbreviations: IMRT, intensity-modulated radiotherapy; SBRT, stereotactic body radiotherapy; CCRT, concurrent chemoradiotherapy.

Data in parentheses are percentages.

### IMRT technique

The techniques of planning and delivery of IMRT were described previously [5]. Briefly, the dose prescribed was 70–76 Gy, 70 Gy, 60 Gy and 50–56 Gy delivered within 6.5 weeks at the periphery of the GTVnx, GTVnd, PTV1 and PTV2, respectively, using the simultaneous integrated boost technique. The total dose of the GTVnx, GTVnd and PTV1 was given in 33 fractions. The total dose of the PTV2 was given in 28–30 fractions at 1.82–1.87 Gy per daily fraction. Two separate plans were made to accomplish the protocol. The inverse IMRT planning system developed by Philips (Madison, WI), either the Pinnacle version 7.4 or version 8.0 planning system was used to do all treatment plans. The IMRT plan mainly consisted of multileaf collimator segments of 6-MV isocentric, coplanar beams arranged in nine almost equally spaced beam angles.

### Boost for residual disease

Residual disease clinically diagnosed on physical examination (including endoscopic examination) and follow-up CT or MRI was treated with boost irradiation. In the CCRT group, 29 received boost treatment after IMRT because of residual disease: 18 patients were treated with IMRT boost with a median dose of 7.08(4.6–15) Gy at 2–3 Gy per daily fraction, and the median time between the end of the primary course radiotherapy to the IMRT boost was 10 (1–45) days; 11 were treated with stereotactic body radiotherapy boost (SBRT) with a median dose of to 15(10–24) Gy at 2.5–4 Gy per fraction and the median time between the end of the primary course radiotherapy to the SBRT boost was 35 (12–80) days. In the IMRT alone group, 16 received boost treatment after IMRT because of residual disease: 9 patients were treated with IMRT boost with a median dose of 7.5(4.48–17.84) Gy at 2–3 Gy per daily fraction, and the median time between the end of the primary course radiotherapy to the IMRT boost was 14 (1–61) days; 7 were treated with SBRT with a median dose of to 15(13.5–24) Gy at 3–4 Gy per fraction and the median time between the end of the primary course radiotherapy to the SBRT boost was 19 (17–26) days.

### Concurrent chemotherapy

Planned concurrent chemotherapy was consisted of weekly intravenous cisplatin at 30 mg/m2 for 7 weeks. Of these 117 patients in the CCRT group, 97 (82.9%) patients received 5 and more cycles of weekly chemotherapy, while 20 (17.1%) patients received 4 cycles or less. The patients in the IMRT alone group did not receive concurrent chemotherapy due to advanced age, heart disease, hepatitis, severe diabetes, inadequate renal function, or patient refusal.

### Treatment monitoring

All patients were evaluated weekly during radiation therapy, and were required to be followed-up after the completion of radiotherapy: 1 month after the completion of radiotherapy, every 3 months in the first 2 years, every 6 months from Year 3 to Year 5, and annually thereafter. Each follow-up included a complete examination that includes flexible fiberoptic endoscopy, ultrasound of abdomen, chest X-ray, and basic serum chemistry. Either CT or MRI of the head and neck was performed after the completion of IMRT and then every 6 months. Treatment induced toxicities were scored according to the Common Terminology Criteria for Adverse Events version 3.0.

### Statistical analysis

The Statistical Package for Social Sciences, version 17.0 (SPSS, Chicago, IL), software was used for statistical analysis. The local failure-free survival (LFFS), regional failure free survival (RFFS), distant failure-free survival (DFFS), and overall survival (OS) were estimated by use of the Kaplan—Meier method. LFFS, RFFS, DFFS, and OS were measured from Day 1 of radiotherapy to the date of the event. Log-rank test was used in univariate analysis. Chi-square, Fisher’s exact, and Student’s t-tests were used to compare the differences between the CCRT group and the IMRT alone group. Multivariate analysis was performed using the Cox proportional hazards model. All statistical tests were two sided, and P < 0.05 was considered to be statistically significant.

## Results

### Outcome of the CCRT Group

The median follow-up time was 58.97 months (range, 5.52–101.26 months). For the patients in the CCRT group, the 1, 3 and 5-year LFFS rates were 97.3%, 88.3% and 86.0%, RFFS rates were 99.1%, 96.3% and 96.3%, DFFS rates were 89.5%, 81.5% and 75.9%, and OS rates were 93.1%, 80.8% and 66.8%, respectively. The most frequently observed acute toxicity was mainly Grade 1 or Grade 2. The incidence of acute Grade 3 mucositis (including pharyngitis), skin reaction, and leukopenia was 21.4%, 10.3% and 12%, respectively (Table 2).

**Table 2:** Frequency of acute toxicity during IMRT.

	CCRT group(N = 117)						IMRT alone group(N = 63)				
	Grade						Grade				P
**Toxicity**	0	1	2	3	4	0	1	2	3	4	
**Mucositis(including pharyngitis)**	0	30(25.6)	62(53.0)	25(21.4)	0	0	17(27.0)	31(49.2)	15(23.8)	0	.882
**Skin reaction**	0	56(47.9)	49(41.9)	12(10.3)	0	1(1.6)	29(46.0)	24(38.1)	8(12.7)	1(1.6)	.392
**Xerostomia**	4(3.4)	83(70.9)	30(25.6)	0	0	3(4.8)	44(69.8)	16(25.4)	0	0	.876
**Anemia**	110(94.0)	7(6.0)	0	0	0	63(100)	0	0	0	0	.098
**Leukopenia**	28(23.9)	43(36.8)	32(27.4)	14(12.0)	0	47(74.6)	13(20.6)	2(3.2)	1(1.6)	0	.000
**Thrombocytopenia**	106(90.6)	8(6.8)	3(2.6)	0	0	62(98.4)	1(1.6)	0	0	0	.155

Data presented as numbers of patients, with percentages in parentheses.

At the last follow-up visit, late toxicities of xerostomia, trismus, subcutaneous tissue fibrosis, otologic toxicities, visual impairment, radiation encephalopathy and hypothyroidism could be evaluated in 58, 64, 65, 64, 63, 69 and 93 patients, respectively. Also, xerostomia were evaluated in 34 patients at 12 months after IMRT. The late radiation toxicity data are reported in Table 3.

**Table 3:** Frequency of late toxicity after IMRT.

	CCRT group					IMRT alone group				
	Grade					Grade				
**Toxicity**	N	0	1	2	3	N	0	1	2	3
**Xerostomia at the last follow-up**	58	5 (8.6)	36(62.1)	17(29.3)	0	30	3(10.0)	18(60.0)	9(30.0)	0
**Trismus**	64	50(78.1)	6(9.4)	8(12.5)	0	42	32(76.2)	5(11.9)	5(11.9)	0
**Subcutaneous tissue fibrosis**	65	17(26.2)	39(60.0)	8(12.3)	1(1.5)	41	8(19.5)	28(68.3)	2(4.9)	3(7.3)
**Otologic toxicities**	64	28(43.8)	-	30(46.9)	6(9.4)	40	14(35.0)	-	21(52.5)	5(12.5)
**Visual impairment**	63	56(88.9)	7(11.1)	0	0	40	35(87.5)	5(12.5)	0	0
**Radiation encephalopathy**	69	57(82.6)	8(11.6)	2(2.9)	2(2.9)	41	36(87.8)	3(7.3)	0	2(4.9)
**Hypothyroidism**	93	46(49.5)	39(41.9)	8(8.6)	0	50	25(50.0)	21(42.0)	4(8.0)	0
**Xerostomia at 12 months after IMRT**	34	0	22(64.7)	12(35.3)	0	30	2(6.7)	18(60.0)	10(33.3)	0

Data presented as numbers of patients, with percentages in parentheses.

### Outcome of the IMRT alone Group

The median follow-up time was 59.24 months (range, 2.79–114.92 months). For the patients in the IMRT alone group, the 1, 3 and 5-year LFFS rates were 98.3%, 91.2% and 85.6%, RFFS rates were 98.3%, 90.6% and 90.6%, DFFS rates were 90.1%, 77.4% and 77.4%, and OS rates were 92.0%, 75.4% and 62.4%, respectively. The most frequently observed acute toxicity was mainly Grade 1 or Grade 2. The incidence of acute Grade 3 mucositis (including pharyngitis), skin reaction, and leukopenia was 23.8%, 12.7% and 1.6%, respectively. One patients (1.6%) had Grade 4 skin reaction (Table 2).

At the last follow-up visit, late toxicities of xerostomia, trismus, subcutaneous tissue fibrosis, otologic toxicities, visual impairment, radiation encephalopathy and hypothyroidism could be evaluated in 30, 42, 41, 40, 40, 41 and 50 patients, respectively. Also, xerostomia were evaluated in 30 patients at 12 months after IMRT(Table 3).

### Outcome of the whole Group

**Fig 1:**
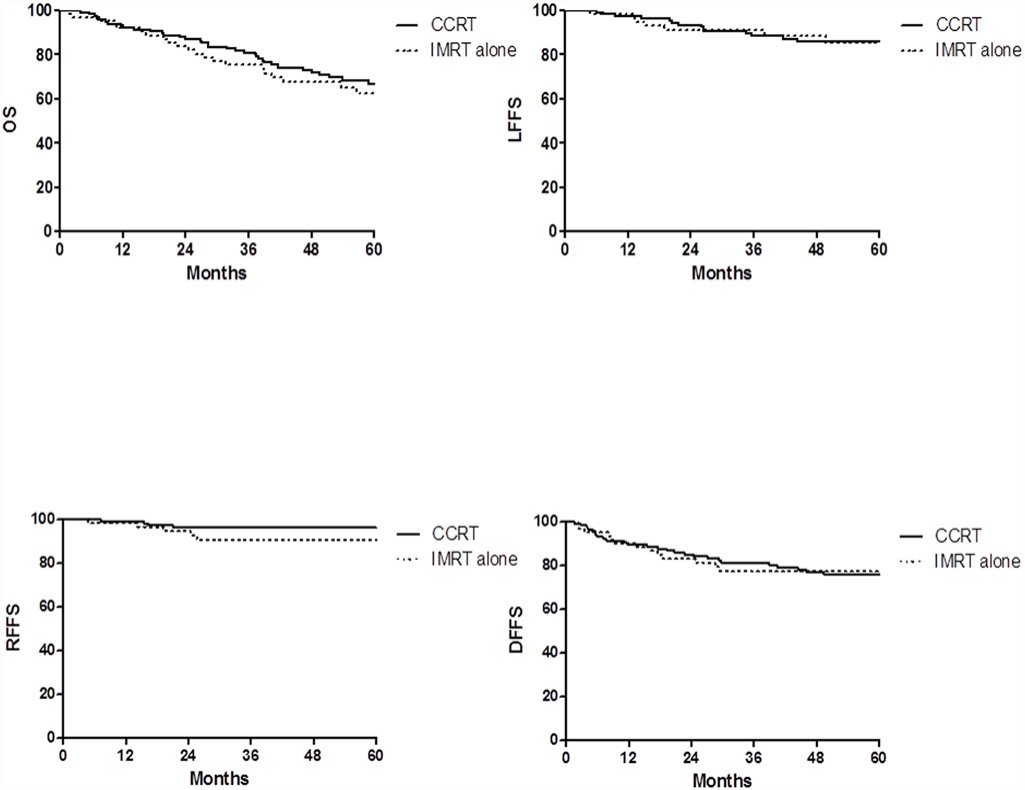
Kaplan-Meier curve showing OS, LRRS, RFFS and DFFS of patients with T4 classification NPC treated with IMRT alone or CCRT in the study.

The median follow-up time was 58.97 months (range, 2.79–114.92) months. For all the patients, the 1, 3 and 5-year LFFS rates were 97.7%, 89.2% and 85.9%, RFFS rates were 98.9%, 94.4% and 94.4%, DFFS rates were 89.7%, 79.9% and 76.2%, and OS rates were 92.7%, 78.9% and 65.3%, respectively. No statistically significant difference was observed in LFFS, RFFS, DFFS and OS between the CCRT group and the IMRT alone group (Fig. 1). No statistically significant difference was observed in acute toxicity except leukopenia (p = 0.000) during IMRT between the CCRT group and the IMRT alone group (Table 2).

### Prognostic factors

The value of various potential prognostic factors including age, gender, N classification, involvement of cranial nerves, volume of GTVnx, metastasis to retropharyngeal lymph nodes, use of boost and CCRT on predicting LFFS, RFFS, DFFS, and OS were evaluated. The outcomes are shown in Tables 4 and 5. In multivariate analysis, CCRT was not an independent factor for LFFS, RFFS, DFFS and OS.

**Table 4:** Impact of prognostic factors on treatment results by univariate analysis.

Items	5y-LFFS		5y-RFFS		5y-DFFS		5y-OS	
	%	*p*	%	*p*	%	*p*	%	*p*
**Gender**								
Male	85.1	.662	93.9	.679	74.6	.433	62.9	.211
Female	88.4		95.5		80.6		73.4	
**Age**								
≥50 y	83.4	.599	94.4	.944	78.6	.645	55.2	.011
<50y	87.9		94.4		74.1		73.9	
**Involvement of cranial nerves**								
NO	89.0	.049	95.8	.176	76.0	.840	71.3	.033
YES	76.9		90.1		77.3		49.3	
**N classification**								
N0–1	87.5	.922	97.0	.227	82.0	.188	74.4	.347
N2–3	84.8		92.5		72.3		59.5	
**Metastasis to (RLN)**								
NO	83.4	.307	97.3	.408	74.5	.971	70.7	.585
YES	86.5		93.5		76.7		63.8	
**Concurrent chemoradiotherapy**								
NO	85.6	.762	90.6	.162	77.4	.788	62.4	.463
YES	86.0		96.3		75.9		66.8	
**Boost**								
NO	87.9	.368	95.0	.478	76.7	.975	66.7	.422
YES	80.4		92.6		74.6		60.7	
**Volume of GTVnx**								
>50 ml	75.2	.004	92.8	.453	78.4	.991	52.8	.008
≤50ml	94.2		95.5		77.2		78.7	

Abbreviations: RLN, retropharyngeal lymph nodes; GTVnx, gross tumor volume of the nasopharynx; LFFS, local failure-free survival; RFFS, regional failure-free survival; DFFS, distant failure-free survival; OS, overall survival.

**Table 5:** Impact of prognostic factors on treatment results by multivariate analysis (*p* value).

Items	5y-LFFS	5y-RFFS	5y-DFFS	5y-OS
**Gender**				
Male vs. Female	.452	.794	.419	.424
**Age**				
≥50 y vs. <50y	.892	.842	.525	.007
**Involvement of cranial nerves**				
NO vs. YES	.330	.200	.939	.183
**N classification**				
N0–1 vs. N2–3	.751	.196	.306	.597
**Metastasis to (RLN)**				
NO vs. YES	.105	.483	.691	.644
**Concurrent chemoradiotherapy**				
NO vs. YES	.669	.086	.875	.974
**Boost**				
NO vs. YES	.355	.656	.847	.403
**Volume of GTVnx**				
>50 ml vs. ≤50ml	.018	.974	.894	.296

Abbreviations: RLN, retropharyngeal lymph nodes; GTVnx, gross tumor volume of the nasopharynx; LFFS, local failure-free survival; RFFS, regional failure-free survival; DFFS, distant failure-free survival; OS, overall survival.

## Discussion

The use of IMRT for NPC was first reported by Sultanem et al.and the updated results were excellent [6,7]. Three randomized trials [4, 8, 9] comparing IMRT versus 2-dimensional technique (2DRT) for NPC have been reported and the trial from Peng et al. indicated that IMRT improved the local-recurrence free survival, nodal relapse-free survival and overall survival. In the IMRT group, the 5-year local control rates of T4 classification NPC were 81.5%, the 5-year overall survival rates of stage IVa and stage IVb were 72.9% and 42.8%, respectively, which is similar to our results.

As IMRT has been accepted as the standard treatment technique for NPC [10], people started to reconsider the role of CCRT. In these two clinical trials of locally or regionally advanced NPC (NPC 9901/9902), 42–52% patients in each group received conformal throughout technique and there were no significant differences in terms of overall survival between the groups of CCRT and RT alone [11,12]. Recently, two large retrospective studies [13, 14] indicated that CCRT failed to improve the prognosis of NPC treated by IMRT.

Compared with 2DRT, IMRT could reduce the radiation-induced toxicities [4]. Also, the trials from Pow et al. and Kam et al. indicated that IMRT for patients with NPC could preserve parotid function and improve corresponding subscale scores on quality of life [8,9]. In the intergroup study 0099 [15] the incidence of Grade 3 or Grade 4 acute toxicity in the radiotherapy alone group and the CCRT group was 50.0% (34/68) and 75.6% (59/78), respectively. A higher incidence of Grade 3 leukopenia was observed in the CCRT group (1/68 vs. 23/78; p <. 05). In our study, the incidence of Grade 3 leukopenia in the CCRT group was higher (12% vs. 1.6%; p = 0.000). As CCRT with high acute treatment-related toxicities in patients who received cisplatin 100 mg/m2 every 3 weeks, the study from Kim et al. [16] indicated that compared with the 3-week cycle of 100mg cisplatin, weekly 30mg cisplatin-based CCRT was a practical, feasible regiment for the patients with locally advanced NPC in regard to decreasing the interruption of radiation treatment and decreasing the treatment-related acute toxicities. In this study of NPC treated by IMRT [13], about 25% patients received neoadjuvant chemotherapy and concurrent chemotherapy was consisted of cisplatin 80 mg/m2 IV every 3 weeks, cisplatin 30–40 mg/m2 IV weekly, or cisplatin 80 mg/m2 IV on day 1 and 5-Fu 800 mg/m2/d continuously IV on day 1–5. Compared with the patients receiving IMRT without concurrent chemotherapy, patients who received concurrent chemotherapy had significantly severer mucositis (Grade 3: 21.5% vs. 43.9%; p < 0.001). In our study, no patients received neoadjuvant chemotherapy and concurrent chemotherapy was consisted of cisplatin 30 mg/m2 IV weekly. No statistically significant difference was observed in acute toxicity except leukopenia between the CCRT group and the IMRT alone group.

In head and neck squamous cell carcinoma, rectal cancer, anal carcinoma and breast cancer treated with radiotherapy [17–21], high-grade acute toxicity was associated with better outcomes. Of interest, no statistically significant difference was found in terms of acute toxicity (not including leukopenia), LFFS, RFFS, DFFS, and OS between the CCRT group and the IMRT alone group in the present study.

There are several limitations in the current study, including the retrospective nature of the study design, the inclusion of patients who completed treatment only and the limited number of patients in the IMRT alone group, which could affect the outcomes. Nevertheless, our report is noteworthy because this is the first study to evaluate concurrent chemotherapy for T4 classification NPC treated by IMRT.

## Conclusions

IMRT alone for T4 classification NPC achieved similar treatment outcomes in terms of disease local control and overall survival as compared to concurrent chemotherapy plus IMRT. However, this is a retrospective study with a limited number of patients, such results need further investigation in a prospective randomized clinical trial.
